# Effect of calcium stearoyl-2 lactylate and lipase supplementation on growth performance, gut health, and nutrient digestibility of broiler chickens

**DOI:** 10.5713/ajas.19.0595

**Published:** 2019-11-12

**Authors:** Samiru Sudharaka Wickramasuriya, Hyun Min Cho, Shemil Priyan Macelline, Eunjoo Kim, Taeg Kyun Shin, Young Joo Yi, Seung Hwan Park, Kyung Bon Lee, Jung Min Heo

**Affiliations:** 1 Department of Animal Science and Biotechnology, Chungnam National University, Daejeon 34134, Korea; 2 Division of Biotechnology, Chonbuk National University, Iksan 54596, Korea; 3 Department of Agricultural Education, College of Education, Sunchon National University, Suncheon 57922, Korea; 4 SNH Biotech Co., Ltd., Daejeon 34008, Korea; 5 Deaprtment of Biology Education, College of Education, Chonnam National University, Gwangju 61186, Korea

**Keywords:** Broiler, Calcium Stearoyl-2 Lactylate, Emulsifier, Growth Performance, Lipase

## Abstract

**Objective:**

To evaluate calcium stearoyl-2 lactylate (CSL) performance as an exogenous emulsifier together with lipase for broiler diets.

**Methods:**

In total, 252 one-day-old Ross 308 broiler chickens were allocated in a completely randomized design to give 6 replications per treatment with 7 birds in each cage. There were six dietary treatments representing a 2×3 factorial arrangement consisted of two energy levels (standard energy [positive control, PC] and −100 kcal/kg of the requirement level [negative control, NC]) and three dietary treatments (without additives [CON], CON+CSL [CSL], and CON+CSL+lipase [CSL-Lipase]). Corn and soybean meal-based experimental diets containing vegetable oil were formulated. Growth performance, blood parameters, visceral organ weights, ileal morphology, nutrient digestibility, and cytokine gene expression were measured.

**Results:**

Birds fed a diet including CSL increased (p<0.05) lipase level in blood compared to birds fed a diet including CSL-Lipase on day 21. Similarly, higher (p<0.05) liver weight was observed in birds fed a diet including either CSL or CSL-Lipase on day 21. Birds fed NC diet with CSL improved (p<0.05) nutrient digestibility compared to the NC diet on day 21. However, birds fed a diet supplemented with CSL or CSL-Lipase did not affect (p>0.05) the weight gain, feed efficiency, ileal morphology, and cytokine concentrations during the experiment period, regardless of dietary energy levels.

**Conclusion:**

Our results indicated that CSL has a role in improving nutrient digestibility in young birds when supplemented to a corn-soybean meal based broiler diet.

## INTRODUCTION

Dietary energy is vital not only for poultry nutrition, but also for the entire animal nutrition as it is a major cost component in diets. Among energy sources, dietary fat and oil support a reasonable level of energy onto diet, and different types of fat can influence growth performance of fast growing broiler chickens (i.e., Ross 308 and Cobb 500; [1,2]). However, hampered fat digestion and absorption from feed matrix were reported in young broiler chickens with an incompletely developed digestive tract [3]. As a dietary tool to improve fat utilization, different emulsifiers such as lysolecithin (lecithin), milk derived casein, soy-lecithin, bile salt, and glycerol polyethylene glycol ricinoleate was tested previously in broiler diets [4]. It may be postulated that exogenous emulsifiers make fat globules more available by optimizing lipase activity and in turn enhancing micelles formation [2,5]. However, limited research conducted with the exogenous emulsifiers and inconsistent results were noted with broiler studies. A couple of studies observed improved growth performance and nutrient digestibility of the broiler chickens [6,7]. Contrary, Cho et al [8] and Zampiga et al [9] reported emulsifier had no such effect on growth performance in broiler chickens. The interaction between the fat type and the activity of the emulsifier might be the source of the observed contrast between studies.

After emulsification, fat start to digest with the aid of bile acid and endogenous lipase action at the lipid-water-interface in the gastrointestinal tract [1,10]. Nevertheless, lower natural lipase production in young birds limit the fat digestion in the early stages of life [11]. In this regard, studies elaborated the importance of adding lipase into broiler diets to improve fat digestion, especially in young broiler chickens.

Calcium stearoyl-2 lactylate is a white or slightly yellowish powder which is widely used in the bakery and dessert industry for food emulsification and stabilization. Commercially, CSL is produced by the esterification process of stearic acid with lactic acid and thereafter it is neutralized to the calcium salts [12,13]. Recently, several studies were conducted with sodium stearoyl-2-lactylate, which is similar with CSL, to evaluate growth performance and nutrient digestibility in broiler chickens [6,14]. Outdated studies have shown the divergent effects of CSL on growth performance, blood parameters, and internal organ of the rats, mouse, and guinea pig [15]. In a meanwhile, no finding was available regarding CSL supplementation on broiler diets and its effect on birds performance.

Consequently, the objective of the present study was to evaluate the effect of CSL and CSL-Lipase on growth performance, blood metabolites, visceral organ weights, ileal morphology, nutrient digestibility, and cytokine gene expression in young broiler chickens fed corn and soybean meal based diet containing vegetable oil from hatch to 28 days.

## MATERIALS AND METHODS

### Animal care

The experiment protocol received prior approval after reviewing from the Animal ethics committee of the Chungnam National University (Protocol No. CNU - 00863).

### Birds, housing and experimental design

Two hundred fifty-two, 1-day-old Ross 308 broiler chickens were used for 28 days experiment. Birds were allocated in a completely randomized design to give six dietary treatments in a 2×3 factorial arrangement. Each dietary treatment consisted of six replicate-pens of seven birds were housed in a raised wire-floor pen (0.85×0.55×0.35 m^3^) with similar body weights (53.13±0.68 g) at the beginning of the experiment. All the management practices were followed Ross 308 broiler management guidance [16].

### Experimental diets and treatments

Corn soybean-meal based diets with two different energy levels (positive control [PC], standard energy vs negative control [NC], 100 kcal/kg deficient energy) were formulated for each feeding phase separately (Table 1) to meet or exceed Ross 308 broiler nutrient specification [17]. All the diets contained the maximum of 4% soybean oil as an energy source. These two diets were fed with or without supplementing either CSL (0.05%; SNH Biotech Co., Ltd., Daejeon, Korea) alone or CSL with lipase (0.05%, SNH Biotech Co., Ltd., Korea) in such manner to produce 6 dietary treatments. Additionally, 0.3% Cr_2_O_3_ (>99.9%, Sigma-Aldrich, St. Louis, MO, USA) was added for all experimental diets as an internal indigestible marker for digestibility analysis. The added lipase was contained 5,000 lipase units per gram as enzyme activity. Birds were offered the experimental diets on an *ad-libitum* basis and had free access to fresh clean drinking water via nipple drinkers throughout the experiment for 28 days.

### Growth performance

Pen basis initial body weights of the birds were recorded, which divided similarly in order to maintain replicate and treatment uniformity of the experiment on day 1. Thereafter, body weight and feed intake were measured on day 7, 14, 21, and 28. Based on the measured body weight and feed intake data, average daily gain, average daily feed intake, and feed conversion ratio were calculated on a pen basis.

### Sample collection

Sample collections were carried out on days 21 and 28. Twelve birds per treatment (two from each replicate which were closest to the median body weight) were selected and the individual body weight recorded and blood samples collected. Blood samples were drawn into BD Vacutainer SST tubes contain polymer gel for serum separation (BD Biosciences, Franklin Lakes, NJ, USA) and BD Vacutainer coated with K2 ethylenediaminetetraacetic acid (BD Biosciences, USA) for leucocyte separation. Thereafter, birds were euthanized by cervical dislocation for following sample collection.

Abdominal incisions were made on each sacrificed bird to separate the ileum from the gastrointestinal tract. The ileum was defined as the segment of small intestine that extended from Meckel’s diverticulum to the ileocecal junction [18]. A 3 cm piece of ileum were removed and flushed with ice-cold phosphate-buffered saline at pH 7.4. The sample was placed into plastic containers contained 10% formaldehyde for fixation and stored until mucosal morphology measurements analysis. Following the separation of morphological samples, the remained digesta of the ileal segment was gently collected by finger stripping into labeled plastic containers for ileal digestibility analysis. Samples were stored in a −20°C freezer until further analysis.

At the same time, liver, spleen, and gizzard were removed separately. Contents and the excess fat were removed manually and the organ weights were recorded [19]. The liver, spleen, and gizzard weights were expressed as proportions of live body weight of the respective bird.

### Sample preparation and analyses

Blood samples collected into vacutainers were then centrifuged (LABOGENE 1248R, Gyrozen Co., Ltd., Daejeon, Korea) to separate serum at 3,000×g for 10 min at 4°C. Separated serum samples were stored at −80°C (UniFreez U 400, DAIHAN Scientific Co., Ltd, Wonju, Korea) for later analysis. Afterward, blood cholesterol, glucose, lipase, and triglyceride levels were analyzed using HITACHI 7180 chemistry analyzer (HITACHI, Tokyo, Japan).

Mononuclear cells were separated from the whole blood using SepMate (STEMCELL Technologies Inc. Vancouver, BC, Canada) tube with Lymphoprep (Axis-Shield PoC AS, Oslo, Norway) solution according to the method described by Stone et al [20]. Harvested peripheral blood mononuclear cells were then cleaned, pelleted and stored at −80°C (UniFreez U 400, Daihan Scientific Co., Ltd, Korea) for analysis.

Total RNA was extracted from lymphocytes using RNAiso plus (Takara, Otsu, Japan) and its concentrations were confirmed by BioSpec-nano (Shimadzu, Kyoto, Japan). Complementary DNA was synthesized using ReverTra Ace qPCR RT kit (Toyobo, Osaka, Japan) after 500 ng of RNA was heated at 65°C for 5 min. The relative gene expressions of interleukin 1 beta (*IL-1β*), IL-6, tumor necrosis factor alpha (*TNF-α*), and interferon gamma (*IFN-γ*) were measured by quantitative real-time polymerase chain reaction (PCR) (StepOnePlus real-time system, ThermoFisher Scientific, Waltham, MA, USA) with SYBR green PCR reagent (TOPreal qPCR 2X Premix, Enzynomics, Daejeon, Korea) and normalized to the level of b-actin as a reference gene. The primers sequences for those genes were presented in Table 2.

Collected ileal samples were processed to make morphological analysis slides as described by Pelicano et al [21]. Ring-shaped lengths of ileum samples were resected, dehydrated, and embedded in paraffin wax. From each of these, six transverse sections (4 to 6 μm) were excised, stained with hematoxylin and eosin, and mounted on glass slides. The morphological measurements (10 measurements from each slide) were taken from well-oriented villi and their associated crypts using NIS-Elements Viewer software (Version: 4.20; NIS Elements, Nikon, Melville, NY, USA) with an inverted microscope (Eclipse TE2000, Nikon Instruments Inc., Melville, NY, USA) using a calibrated eyepiece graticule.

Nutrient digestibility analysis was performed according to the method described by Huang et al [22] with slight modifications. Dry matter, crude protein, and gross energy of the feed and digesta samples were analysed according to AOAC [23] methods. Chromium oxide contents were analysed according to Fenton and Fenton [24] method. Calculations were made for apparent digestibility values for nutrients as follows [25,26].

$$\text{Digestibility }(\%)=100-\frac{\text{ID}}{\text{IF}}\times \frac{\text{AF}}{\text{AD}}$$

Where, ID denotes the concentration of an indigestible marker in the diet; IF is the indigestible-marker concentration in ileal digesta; AF is the nutrient concentration in ileal digesta, and AD is the nutrient concentration in the diet.

### Statistical analyses

Obtained data were analyzed as a 2×3 factorial arrangement utilizing SPSS software (Version 21; IBM SPSS 2012). Two way analysis of variance in general linear model procedure was performed to determine the main effects of dietary energy levels, emulsifier supplementation and their interactions. A pen was used as the experimental unit for all growth performance measurements. Selected individual birds were considered the experimental unit for blood parameters, visceral organ weights, ileal morphology, nutrient digestibility, and blood cytokine concentrations, respectively. Mean differences observed in the treatment were considered significant at p<0.05. When treatment effects were significant (p<0.05), means were separated using Duncan multiple range test procedures of SPSS software (Version 24; IBM SPSS, 2016).

## RESULTS

The effect of the dietary energy levels and the emulsifier addition on daily gain is described in Table 3. No interaction was observed between dietary energy level and emulsifier supplementation (p>0.05) with respect to the daily gain of the broiler chickens from hatch to 28 days of age. Birds fed standard energy level diet increased (p<0.05) average daily gain compared to their counterparts on day 7.

An interaction (p<0.05) was observed between dietary energy levels and emulsifier supplementation on feed intake in broiler chickens on day 28 and the entire period from 1 to 28 days (Table 4). Birds fed NC with CSL had higher (p<0.05) feed intake compared to broiler chickens fed PC including CSL.

No difference (p>0.05) was found in feed efficiency of broiler chickens among dietary energy levels, emulsifier supplementation or their interaction from hatch to 28 days (Table 5).

No interaction or the main effect (p>0.05) was observed from dietary energy levels and emulsifier supplementation on blood parameters on days 21 and 28 except blood lipase (Table 6). On day 21, birds fed a diet including CSL-Lipase reduced (p<0.05) blood lipase level compared to those fed a diet including CSL, regardless of dietary energy levels.

Birds fed CSL or CSL-Lipase increased (p<0.05) the liver weight compared to the birds fed CON on day 21, independent of dietary energy levels (Table 7). Moreover, higher (p< 0.05) spleen weight was observed birds fed standard energy diets, regardless of emulsifier supplementation on day 28. Nevertheless, there were no interaction effect or main effect (p>0.05) from emulsifier supplementation together with dietary energy levels on gizzard weight of the birds on days 21 and 28.

The effect of dietary energy levels and emulsifier supplementation on gut morphology is presented in Table 8. No main effect and interaction were found (p>0.05) in villus height (V), crypt depth (C), and V:C ratio of the broiler chickens on day 21 and 28.

Dietary energy levels and emulsifier supplementation had an interactive effect (p<0.05) on nutrient digestibility of the broiler chickens on day 21 (Table 9). Birds fed NC with CSL had higher (p<0.05) dry matter, protein, and energy digestibility compared to those fed NC treatment on day 21. Nevertheless, no interaction (p>0.05) was observed in nutrient digestibility on day 28. Moreover, birds fed low energy diet had lower (p<0.05) nutrient digestibility, independent of emulsifier supplementation on day 28.

Gene expression of *IL-1β*, *IL-6*, *TNF-α*, and *IFN-γ* were evaluated in broiler chickens fed a diet supplemented with emulsifier together with two dietary energy levels on days 21 and 28 (Table 10). The gene expression of *IL-1β* was shown to be confounding variations among each replicate and between treatments in day 28 and therefore excluded from further analysis. Birds fed a diet including either CSL or CSL-Lipase had lower (p<0.05) *IL-1β* expression compared to birds fed a CON diet on day 21, regardless of dietary energy levels. Nevertheless, no main effect or interaction between dietary energy level and emulsifier supplementation (p>0.05) were observed with IL-6, TNF-α, and IFN-γ expression in broiler chickens on day 21 and 28.

## DISCUSSION

Better utilization of the energy in the diet became a center of interest for the nutritionist during last few decades as a means of reducing the feed cost. In recent years, the addition of fat into practical diet formulation became popular with the increased cereal grain price. However, it has been reported that alone endogenous emulsifiers did not support proper fat digestion in the poultry gut [27]. Exogenous emulsifiers are capable of improving fat digestibility and subsequently sustaining or enhancing growth performance of the broiler chickens fed a low-density energy diet [28]. With this conviction, studies tested different exogenous emulsifiers to improve the performance of the broiler chickens [7,14,29]. Our current study tested the hypotheses that emulsifiers will improve or sustain growth performance with enhanced gut integrity and nutrient digestibility of the birds fed low energy diet. Previous reports indicated that fatty acids components of the vegetable oil source are highly digestible and more sensitive to exogenous emulsifiers compared to animal fat (i.e., lard and tallow; [2,5]. Vegetable oil was selected as the fat source in the present study aiming to achieve a better response from the broiler chickens fed a diet with emulsifiers. Instead of emulsifiers, it also known that dietary fat utilization efficacy depends on lipase activity of broiler chickens [3]. Lipase hydrolyzes triglycerides into fatty acids and promotes the formation of micelles for fat digestion and absorption through the intestinal mucosa [30]. However, it was reported, low natural lipase production and lipase activity limit the fat digestion in young birds [11]. In this light, lipase was added together with CSL as a treatment in our study to comprehend its activity on broiler chicken performance.

In our study, a diet including CSL or CSL-Lipase did not affect growth performance or feed efficiency of broiler chickens compared to those fed CON diet, independent of energy levels. Consistent with our observed results, Zampiga et al [9] did not observe growth performance enhancement in broiler chickens fed a diet with lysophospholipids emulsifier for 42 days. Further, sodium stearoyl-2-lactylate supplemented as an emulsifier did not show growth and feed efficiency improvement in broiler chickens from hatch to 35 days [8]. These superficial growth performances can be ascribed to the notion of low lipase activity and limited bile secretion of the young birds [3,2]. Although no growth improvement was observed in birds fed a diet including CSL-Lipase, the present study confirmed that secretion of exogenous lipase is less dramatic when calculated per gram of feed intake although net secretion increase with the birds’ age [31]. One another possible reason for observed no growth difference in this study may be insufficient inclusion levels of CSL and lipase with their lower activities. Observed interaction effect of feed intake on day 28 and commensurate with the overall period (day 1 to 28) was surprising and underline that the mechanism is unclear in the current study.

Similar to growth performance, blood cholesterol, glucose, and triglycerides levels were not affected in birds fed a diet including CSL or CSL-Lipase. These results are consistent with those of Guerreiro Neto et al [2] who also did not observed differences in blood parameters (i.e., total cholesterol, high-density lipoproteins, and triglycerides) in broiler chickens fed a diet with exogenous emulsifiers. A surprising effect of CSL and CSL-Lipase on the blood lipase level of broiler chickens was observed on day 21. Broiler chickens fed a diet including CSL-Lipase had lower blood lipase levels compared to those fed a diet including CSL, independent of dietary energy levels.

According to previous studies, emulsifier supplementation into diet did not affect the visceral organ weight of broiler chickens [2,8,26,29]. With partial agreement, emulsifier supplementation or energy levels did not influence the proportion of gizzard weight in the present study. Interestingly, birds fed a diet supplemented with CSL or CSL-Lipase showed the higher proportion of liver weight compared to those fed CON diet on day 21. Supporting this observation, higher liver weight associated with exogenous emulsifier also were reported for broiler chickens and ducks [32,28]. The exact reason for observed higher liver weight is unknown and unclear up to date. Perhaps higher liver weight could be attributed to elevated lipid metabolism in the liver as a result of CSL on day 21. Another possible mechanism for this enlarged liver may be the lipogranulomata condition which may derive from acute toxicity of CSL. However, a thorough study of the liver pathology is warranted to reach a confirmatory conclusion in this regard.

Dietary influence and associated intestinal functions are interrelated with the intestinal epithelium morphology of the broiler chickens [14,32]. In this light, we observed ileal morphology indices of the broiler chickens in this study. Nevertheless, ileal morphology in the present study did not differ among dietary treatments. This observation is consistent with those of Zosangpuii et al [32], who reported emulsifier did not affect intestinal villi height of the 42 days old Khaki Campbell ducks. Contrary, Alzawqari et al [33], observed improved gut morphology indices in broiler chickens fed a diet supplemented tallow with desiccated ox bile as a natural emulsifier. A diet supplemented tallow, inclusion rate of desiccated ox bile and natural source of emulsifier might be the reasons for different observation between those two experiments.

Broiler chickens fed NC with CSL showed improved dry matter, crude protein, and energy digestibility compared to those of the other treatments on day 21. Observed diet directed interaction on day 21 emphasized the fact that low energy diet with added emulsifier would improve nutrient digestibility of young broiler chickens. Further, the notion of lower nutrient digestion and absorption with an incompletely developed digestive tract of young broiler chickens was manifested with observed no difference in nutrient digestibility on day 28 in this present study. With the agreement of our observed results, Abbas et al [26] and Siyal et al [28] reported improved nutrient digestibilities in broiler chickens with exogenous emulsifiers. Interestingly, Siyal et al [28] observed more prominent nutrient digestibility in broiler chickens fed a diet containing a higher dosage of the emulsifier compared to those fed no or less dosage. In the present study, broiler chickens fed low energy diet showed low nutrient digestibility compared to standard energy diet, independent of emulsifier supplementation on days 21 and 28.

Cytokines are proteins which involved in the regulation of both innate and adaptive immune response to pathogens [34]. In this study, no difference of IL-6, TNF-α, and IFN-γ expression observed in broiler chickens fed a diet with CSL or lipase addition validated the good management practices followed during the experiment and the absence of any infections to birds. Moreover, it suggests that CSL or CSL-Lipase did not affect immune homeostasis and prevent immune system activation. Nevertheless, lower IL-1β activity in the broiler chickens fed a diet including CSL and CSL-Lipase is confounding and needs be elucidated.

To this end, our result indicated that CSL has a ability to improve nutrient digestibility in young broilers chickens when supplemented into the vegetable oil-based low energy diet at the level of 0.05%. Nevertheless, higher levels of CSL inclusion together with different fat sources are important topics for future research with the aim of achieving better growth performances.

## Figures and Tables

**Table 1:** Ingredient and calculated nutrient composition of experimental diets (g/kg, as-fed basis)

Items	Positive control		Negative control	
	
	1–21 d	22–28 d	1–21 d	22–28 d
Ingredient				
Corn	48.00	59.00	43.28	54.50
Wheat bran	4.17	-	9.79	5.87
Soybean meal	39.60	32.97	38.70	31.60
Soybean oil	4.00	4.00	4.00	4.00
Limestone	1.50	1.50	1.50	1.50
Monocalcium phrousphus	1.70	1.50	1.70	1.50
Iodized salt	0.35	0.30	0.35	0.30
Vit-Min premix1)	0.30	0.30	0.30	0.30
Lys-HCl	0.10	0.14	0.10	0.14
DL-methionine	0.28	0.29	0.28	0.29
Calculated values2)				
ME (kcal/kg)	3,050	3,200	2,950	3,099
CP (%)	22.28	19.71	22.32	19.60
Cfat (%)	6.29	6.43	6.30	6.44
Ca (%)	1.09	1.02	1.10	1.03
Available P (%)	0.49	0.43	0.50	0.44
Lys (%)	1.36	1.20	1.36	1.19
Met (%)	0.62	0.60	0.62	0.60
Met+Cys (%)	1.00	0.94	1.01	0.94
Thr (%)	0.87	0.76	0.86	0.72
Trp (%)	0.28	0.24	0.28	0.24
Val (%)	1.03	0.91	1.03	0.90
Arg (%)	1.48	1.26	1.49	1.26

ME, metabolizable energy; CP, crude protein; Cfat, crude fat.

1) Supplied per kilogram of total diets: Fe (FeSO_4_·H_2_O), 80 mg; Zn (ZnSO_4_·H_2_O), 80 mg; Mn (MnSO_4_·H_2_O) 80 mg; Co (CoSO_4_·H_2_O) 0.5 mg; Cu (CuSO_4_·H_2_O) 10 mg; Se (Na_2_SeO_3_) 0.2 mg; I, (Ca(IO_3_)·2H_2_O) 0.9 mg; vitamin A, 24,000 IU; vitamin D_3_, 6,000 IU; vitamin E, 30 IU; vitamin K, 4 mg; thiamin, 4 mg; riboflavin, 12 mg; pyridoxine, 4 mg; folacine, 2mg; biotin, 0.03 mg; vitamin B_8_, 0.06 mg; niacin, 90 mg; pantothenic acid, 30 mg.

2) The values were calculated according to the values of feedstuffs in NRC (1994).

**Table 2:** The Primers for quantitative real-time polymerase chain reaction

Cytokines		Primer sequence	Gene Bank accession
IL-1β	Forward	5′-ATTACCGTCCCGTTGCTTT-3′	NM_204524
	Reverse	5′-AGTCACAATAAATACCTCCACCC-3′	
IL-6	Forward	5′-AAATCCCTCCTCGCCAATC-3′	NM_204628
	Reverse	5′-CCCTCACGGTCTTCTCCA-3′	
TNF-α	Forward	5′-AGATGGGAAGGGAATGAACC-3′	NM_204267
	Reverse	5′-GAACTGGGCGGTCATAGAA-3′	
IFN-γ	Forward	5′-CAAGTCAAAGCCGCACATC-3′	NM_205149
	Reverse	5′-TTCACCTTCTTCACGCCATC-3′	
β-Actin	Forward	5′-TTACCAACACCCACACCC-3′	L08165
	Reverse	5′-ACACCTTCACCATTCCAGTT-3′	

IL-1β, interleukin 1 beta ; IL-6, interleukin 6; TNF-α, tumor necrosis factor alpha; IFN-γ, interferon gamma; β-Actin, beta actin.

**Table 3:** Effects of dietary energy levels, emulsifier and lipase supplementation in a diet on daily gain (g/d) of broiler chickens

Dietary treatments1)		Day 7	Day 14	Day 21	Day 28	Day 1–21	Day 1–28
PC		21.79	50.30	66.02	86.26	46.04	56.09
PC+CSL		21.07	50.58	64.78	90.82	45.48	56.81
PC+CSL+Lipase		21.65	50.34	65.38	91.05	45.79	57.11
NC		19.86	48.34	63.64	89.66	43.95	55.37
NC+CSL		20.82	49.64	67.34	80.71	45.94	54.63
NC+CSL+Lipase		20.51	49.34	65.28	88.75	45.04	55.97
SEM		0.190	0.392	0.736	1.767	0.266	0.506
Main effect means							
Energy2)	Standard	21.50	50.41	65.39	89.38	45.77	56.67
	Low	20.40	49.10	65.42	86.37	44.97	55.32
Emulsifier3)	CON	20.82	49.32	64.83	87.96	44.99	55.73
	CSL	20.95	50.11	66.06	85.77	45.71	55.72
	CSL+Lipase	21.08	49.84	65.33	89.90	45.41	56.54
p-values	Energy	0.007	0.108	0.985	0.402	0.147	0.193
	Emulsifier	0.861	0.706	0.792	0.638	0.550	0.755
	Interaction	0.208	0.838	0.401	0.307	0.165	0.831

SEM, standard error of mean.

1) PC (positive control), standard energy control diet; NC (negative control), low energy control diet; CSL, calcium stearoyl-2 lactylate.

2) Values are the mean of 18 replicates per treatment.

3) Values are the mean of 12 replicates per treatment.

**Table 4:** Effects of dietary energy levels, emulsifier and lipase supplementation in a diet on daily feed intake (g/d) of broiler chickens

Dietary treatments1)		Day 7	Day 14	Day 21	Day 28	Day 1–21	Day 1–28
PC		29.43	65.01	91.99	144.92^ab^	62.14	85.95^ab^
PC+CSL		31.69	67.16	96.68	133.88^a^	65.18	82.36^a^
PC+CSL+Lipase		32.22	64.68	97.93	144.04^ab^	64.95	84.72^ab^
NC		31.96	65.40	96.73	147.86^ab^	64.69	86.12^ab^
NC+CSL		31.41	67.36	95.08	160.48^b^	64.61	91.99^b^
NC+CSL+Lipase		31.32	66.90	96.15	138.32^ab^	64.79	86.71^ab^
SEM		0.370	0.795	0.810	2.108	0.417	0.711
Main effect means							
Energy2)	Standard	31.11	65.61	95.54	140.95	64.09	84.34
	Low	31.56	66.55	95.98	148.89	64.70	88.28
Emulsifier3)	CON	30.69	65.20	94.36	146.39	63.42	86.04
	CSL	31.55	67.26	95.88	147.18	64.89	87.18
	CSL+Lipase	31.77	65.79	97.04	141.18	64.87	85.72
p-values	Energy	0.551	0.559	0.785	0.069	0.471	0.010
	Emulsifier	0.466	0.561	0.410	0.460	0.270	0.682
	Interaction	0.148	0.849	0.191	0.011	0.270	0.025

SEM, standard error of mean.

1) PC (positive control), standard energy control diet; NC (negative control), low energy control diet; CSL, calcium Stearoyl-2 lactylate.

2) Values are the mean of 18 replicates per treatment.

3) Values are the mean of 12 replicates per treatment.

a,b Means in the same column with different superscripts differ (p<0.05).

**Table 5:** Effects of dietary energy levels, emulsifier and lipase supplementation in a diet on feed efficiency of broiler chickens

Dietary treatments1)		Day 7	Day 14	Day 21	Day 28	Day 1–21	Day 1–28
PC		1.48	1.35	1.45	1.67	1.43	1.53
PC+CSL		1.53	1.35	1.44	1.67	1.44	1.50
PC+CSL+Lipase		1.57	1.31	1.50	1.63	1.47	1.51
NC		1.47	1.30	1.47	1.73	1.42	1.50
NC+CSL		1.49	1.33	1.47	1.78	1.44	1.57
NC+CSL+Lipase		1.45	1.33	1.47	1.52	1.42	1.50
SEM		0.019	0.015	0.015	0.034	0.009	0.016
Main effect means							
Energy2)	Standard	1.53	1.34	1.46	1.66	1.44	1.51
	Low	1.47	1.32	1.47	1.68	1.42	1.52
Emulsifier3)	CON	1.48	1.32	1.46	1.70	1.42	1.52
	CSL	1.51	1.34	1.46	1.72	1.44	1.53
	CSL+Lipase	1.51	1.32	1.49	1.58	1.44	1.50
p-values	Energy	0.153	0.618	0.762	0.771	0.262	0.683
	Emulsifier	0.715	0.794	0.667	0.199	0.642	0.683
	Interaction	0.476	0.671	0.620	0.387	0.543	0.326

SEM, standard error of mean.

1) PC (positive control), standard energy control diet; NC (negative control), low energy control diet; CSL, calcium stearoyl-2 lactylate.

2) Values are the mean of 18 replicates per treatment.

3) Values are the mean of 12 replicates per treatment.

**Table 6:** Effects of dietary energy levels, emulsifier and lipase supplementation in a diet on the blood profile of broiler chickens

Dietary treatments1)		Day 21				Day 28			
	
		Cholesterol (mg/dL)	Glucose (mg/dL)	Lipase (U/L)	TG (mg/dL)	Cholesterol (mg/dL)	Glucose (mg/dL)	Lipase (U/L)	TG (mg/dL)
PC		143.58	292.90	10.77	61.72	125.90	275.35	11.42	58.73
PC+CSL		144.53	299.91	12.63	84.25	132.65	275.58	11.22	56.98
PC+CSL+Lipase		132.00	280.40	11.00	68.70	142.65	305.77	11.52	57.33
NC		140.90	301.40	11.85	83.80	125.62	287.32	12.17	54.57
NC+CSL		136.68	275.05	12.88	72.78	132.48	286.24	11.77	68.83
NC+CSL+Lipase		138.95	298.69	10.70	78.27	136.42	292.78	12.27	60.32
SEM		2.669	3.845	0.301	4.481	3.211	3.590	0.262	3.375
Main effect means									
Energy2)	Standard	140.04	291.07	11.46	71.56	133.73	285.57	11.38	57.68
	Low	138.84	291.71	11.81	78.28	131.51	288.78	12.07	61.24
Emulsifier3)	CON	142.24	297.15	11.31^ab^	72.76	125.76	281.33	11.79	56.65
	CSL	140.60	287.48	12.75^b^	78.52	132.57	280.91	11.49	62.91
	CSL+Lipase	135.48	289.55	10.85^a^	73.48	139.53	299.27	11.89	58.83
p-values	Energy	0.825	0.934	0.569	0.459	0.731	0.658	0.202	0.602
	Emulsifier	0.543	0.559	0.048	0.860	0.232	0.074	0.811	0.746
	Interaction	0.527	0.085	0.623	0.336	0.908	0.294	0.984	0.629

TG, triglycerides; SEM, standard error of mean.

1) PC (positive control), standard energy control diet; NC (negative control), low energy control diet; CSL, calcium Stearoyl-2 lactylate.

2) Values are the mean of 18 replicates per treatment.

3) Values are the mean of 12 replicates per treatment.

a,b Means in the same column in the same parameter with different superscripts differ (p<0.05).

**Table 7:** Effects of dietary energy levels, emulsifier and lipase supplementation in a diet on the proportion1) of visceral organ weights of broiler chickens

Dietary treatments2)		Day 21			Day 28		
	
		Liver (%)	Spleen (%)	Gizzard (%)	Liver (%)	Spleen (%)	Gizzard (%)
PC		2.58	0.08	1.57	2.55	0.11	1.03
PC+CSL		3.12	0.08	1.69	2.48	0.12	1.09
PC+CSL+Lipase		2.72	0.08	1.51	2.39	0.10	1.12
NC		2.56	0.08	1.41	2.30	0.09	1.21
NC+CSL		2.86	0.09	1.66	2.52	0.10	1.11
NC+CSL+Lipase		2.83	0.10	1.77	2.54	0.09	1.06
SEM		0.040	0.004	0.039	0.047	0.101	0.031
Main effect means							
Energy3)	Standard	2.80	0.08	1.59	2.47	0.11	1.08
	Low	2.75	0.09	1.61	2.45	0.09	1.13
Emulsifier4)	CON	2.57^a^	0.08	1.49	2.42	0.10	1.12
	CSL	2.99^c^	0.08	1.67	2.50	0.11	1.10
	CSL+Lipase	2.77^b^	0.09	1.64	2.46	0.09	1.09
p-values	Energy	0.496	0.284	0.783	0.846	0.048	0.458
	Emulsifier	0.001	0.613	0.144	0.822	0.250	0.942
	Interaction	0.186	0.761	0.100	0.227	0.784	0.293

SEM, standard error of mean.

1) Organ weights are express as a proportion of live body weight.

2) PC (positive control), standard energy control diet; NC (negative control), low energy control diet; CSL, calcium Stearoyl-2 lactylate.

3) Values are the mean of 18 replicates per treatment.

4) Values are the mean of 12 replicates per treatment.

a–c Means in the same column in the same parameter with different superscripts differ (p<0.05).

**Table 8:** Effects of dietary energy levels, emulsifier and lipase supplementation in a diet on the ileal morphology of broiler chickens

Dietary treatments1)		Day 21			Day 28		
	
		Villus height (μm)	Crypt depth (μm)	V:C ratio	Villus height (μm)	Crypt depth (μm)	V:C ratio
PC		529.81	62.63	10.31	457.21	62.04	7.63
PC+CSL		549.31	60.80	10.85	471.75	60.69	7.98
PC+CSL+Lipase		574.28	51.69	11.73	464.12	63.64	7.56
NC		537.16	61.29	9.00	481.89	68.99	7.28
NC+CSL		597.69	51.99	11.85	442.06	65.02	7.13
NC+CSL+Lipase		575.12	56.65	10.89	424.76	60.22	7.19
SEM		10.680	2.427	0.395	9.873	1.129	0.134
Main effect means							
Energy2)	Standard	551.13	58.38	10.96	464.36	62.13	7.72
	Low	569.99	56.64	10.58	449.57	64.75	7.20
Emulsifier3)	CON	533.48	61.96	9.65	469.55	65.52	7.46
	CSL	573.50	56.40	11.35	456.91	62.86	7.55
	CSL+Lipase	574.70	54.17	11.31	444.44	61.93	7.37
p-values	Energy	0.384	0.724	0.629	0.460	0.255	0.060
	Emulsifier	0.217	0.413	0.153	0.589	0.416	0.865
	Interaction	0.620	0.518	0.459	0.373	0.167	0.697

V:C; villus height:crypt depth; SEM, standard error of mean.

1) PC (positive control), standard energy control diet; NC (negative control), low energy control diet; CSL, calcium stearoyl-2 lactylate.

2) Values are the mean of 18 replicates per treatment.

3) Values are the mean of 12 replicates per treatment.

**Table 9:** Effects of dietary energy levels, emulsifier and lipase supplementation in a diet on the nutrient digestibility of broiler chickens

Dietary treatments1)		Day 21			Day 28		
	
		DM	CP	Energy	DM	CP	Energy
PC		81.17^a^	88.70^a^	83.75^a^	85.37	89.49	87.67
PC+CSL		79.32^a^	87.18^a^	81.69^a^	75.52	80.65	78.63
PC+CSL+Lipase		85.32^a^	90.46^a^	86.95^a^	85.00	89.34	87.09
NC		63.49^b^	75.14^b^	66.23^b^	71.38	80.17	73.17
NC+CSL		77.88^a^	85.85^a^	79.80^a^	72.77	80.17	75.65
NC+CSL+Lipase		77.73^a^	85.22^a^	79.42^a^	77.82	81.79	79.40
SEM		1.170	0.826	1.193	1.551	1.313	1.426
Main effect means							
Energy2)	Standard	81.94	88.78	84.13	81.97	86.49	84.46
	Low	73.03	82.07	75.15	73.99	80.71	76.07
Emulsifier3)	CON	72.33^b^	81.92^b^	74.99^b^	78.38	84.83	80.42
	CSL	78.60^ab^	86.52^ab^	80.75^ab^	74.15	80.41	77.14
	CSL+Lipase	81.53^a^	87.84^a^	83.19^a^	81.41	85.56	83.25
p-values	Energy	0.001	0.001	0.001	0.016	0.036	0.007
	Emulsifier	0.013	0.021	0.031	0.178	0.240	0.234
	Interaction	0.032	0.020	0.044	0.344	0.362	0.270

DM, dry matter; CP, crude protein; SEM, standard error of mean.

1) PC (positive control), standard energy control diet; NC (negative control), low energy control diet; CSL, calcium Stearoyl-2 lactylate.

2) Values are the mean of 18 replicates per treatment.

3) Values are the mean of 12 replicates per treatment.

a,b Means in the same column with different superscripts differ (p<0.05).

**Table 10:** Effects of dietary energy levels, emulsifier and lipase supplementation on blood cytokine concentration

Dietary treatment1)		Day 21				Day 28		
	
		IL-1β	IL-6	TNF-α	IFN-γ	IL-6	TNF-α	IFN-γ
PC		3.28	0.63	2.67	0.18	2.00	2.03	1.98
PC+CSL		0.42	1.12	1.91	1.54	2.67	1.84	6.36
PC+CSL+Lipase		1.15	0.61	2.13	0.35	3.01	3.39	5.74
NC		1.75	1.02	1.79	0.40	1.43	1.15	3.37
NC+CSL		1.11	0.99	3.63	0.69	0.56	1.37	1.74
NC+CSL+Lipase		0.77	1.82	1.90	1.49	2.60	2.06	4.50
SEM		0.235	0.177	0.386	0.268	0.522	0.250	0.949
Main effect means								
Energy2)								
	Standard	1.62	0.79	2.23	0.69	2.56	2.42	4.69
	Low	1.21	1.28	2.44	0.86	1.53	1.53	3.20
Emulsifier3)	CON	2.52^b^	0.83	2.23	0.29	1.72	1.59	2.68
	CSL	0.77^a^	1.06	2.77	1.12	1.61	1.60	4.05
	CSL+Lipase	0.96^a^	1.21	2.02	0.92	2.81	2.73	5.12
p-values	Energy	0.406	0.189	0.795	0.759	0.343	0.099	0.453
	Emulsifier	0.020	0.674	0.722	0.444	0.600	0.147	0.520
	Interaction	0.198	0.328	0.390	0.353	0.769	0.788	0.479

IL-1β, interleukin 1 beta; IL-6, interleukin 6; TNF-α, tumor necrosis factor alpha; IFN-γ, interferon gamma; SEM, standard error of mean.

1) PC (positive control), standard energy control diet; NC (negative control), low energy control diet; CSL, calcium Stearoyl-2 lactylate.

2) Values are the mean of 18 replicates per treatment.

3) Values are the mean of 12 replicates per treatment.

a,b Means in the same column with different superscripts differ (p<0.05).
